# Comparative analysis of mutational robustness of the intrinsically disordered viral protein VPg and of its interactor eIF4E

**DOI:** 10.1371/journal.pone.0211725

**Published:** 2019-02-14

**Authors:** Jocelyne Walter, Justine Charon, Yihua Hu, Joy Lachat, Thomas Leger, Guillaume Lafforgue, Amandine Barra, Thierry Michon

**Affiliations:** 1 UMR Biologie du Fruit et Pathologie, INRA, Université de Bordeaux, CS, Villenave d’Ornon, France; 2 School of Life & Environmental Sciences, The University of Sydney, Sydney, NSW, Australia; University of Valencia, SPAIN

## Abstract

Conformational intrinsic disorder is a feature present in many virus proteins. Intrinsically disordered regions (IDRs) have weaker structural requirement than ordered regions and mutations in IDRs could have a lower impact on the virus fitness. This could favor its exploration of adaptive solutions. The potyviral protein VPg contains IDRs with determinants for adaptation to its host plant. To experimentally assess whether IDRs are more resistant to mutations than ordered regions, the biologically relevant interaction between mutant libraries of both VPg and the eukaryotic translation initiation factor 4E (eIF4E) and their respective wild type partner was examined using yeast two hybrid assay. Our data shows that VPg is significantly more robust to mutations than eIF4E and as such belongs to a particular class of intrinsically disordered proteins. This result is discussed from the standpoint of IDRs involvement in the virus adaptive processes.

## Introduction

Conformational intrinsic disorder is now recognized as a functional feature of a large number of proteins. The observation that protein or protein regions lacking stable and unique 3D conformations [1] carry important biological functions has revolutionized the old and well-established protein structure-function dogma. Since the birth in the early 2000s of the concept of protein intrinsic disorder, numerous studies have highlighted its ubiquitous and multi-functional nature, for review see [2,3]. The RNA virus proteome is particularly enriched in intrinsically disordered regions, IDRs [4,5]. Because of their structural plasticity, IDRs are multifunctional. Notably, many IDRs are involved in multi-partnership, thereby developing between the virus and its host, a complex interactome [6]. A common trait of RNA viruses resides in their extraordinary ability to adapt to fast-changing environments, by creating a high degree of genetic diversity in viral populations. This genetic diversity both result from the unparalleled mutation rates observed in viruses, and their ability to evolve by maintaining mutations in populations, *i*.*e* mutational robustness [7,8]. Mutational robustness is defined as the ability of an organism to maintain its phenotype despite the disturbances caused by mutation accumulation. In case of viruses, this mutational robustness is the result of combined multi-scale processes including functional complementation between individual variants in the population (quasi-species) but also mutation buffering at the molecular level. Indeed, non-conservative mutations within viral proteins often lead to an increased instability, partly buffered by an enhanced chaperone assistance [9]. Another and probably more frequent factor accounting for mutational robustness could reside in intrinsic structural properties of viral proteins [10]. We previously proposed that IDRs could contribute to virus adaptive potential [11], and recently, we reported the first experimental evidence for the involvement of a viral protein intrinsically disordered region in the adaptation of a plant RNA virus to its host [12]. An earlier study was aimed at comparing the structural features of 123 single-domain small-proteins from hypothermophylic bacteria, archaea, mesophilic eukaryota and prokaryota, and RNA or DNA viruses, whose crystal structures were available [13]. It was concluded from this analysis that viral proteins and more particularly RNA virus proteins, display (i) higher stability upon simulations of mutation accumulation (lower ΔΔG per residues compared to the other groups of proteins) and (ii) lower inter-residues contact densities. This latter feature is a typical signature of intrinsic disorder. It has thus been proposed that the large intrinsic disorder content in viral protein could constitute a viral strategy to efficiently buffer mutation effects [13,14], strongly contrasting with non-additive/epistatic stability loss profile expected from ordered proteins as previously reported for a bacterial β-lactamase [15].

The study presented here aimed at experimentally assess if intrinsic disorder within viral proteins is more prone to mutational robustness than ordered proteins. To address this issue, we used the biologically relevant interaction between the viral genome-linked protein (VPg), an intrinsically disordered protein from the potyvirus *Potato virus Y* (PVY), and one of its ordered partner, the eukaryotic translation initiation factor 4E (eIF4E) from pepper *Capsicum annuum* [16]. The VPgs from several potyviruses, namely Lettuce mosaic virus (LMV) [17], Potato virus Y (PVY) [18] and Potato virus A (PVA) [19] have been experimentally characterized as intrinsically disordered. With the exception of its 45 amino acid N-terminal domain, which does not participate in the interaction with VPg [20] [21], eIF4E is an ordered globular protein [22–24]. We compared the mutational robustness of these two proteins subject to random mutagenesis, by monitoring the loss of their respective ability to interact with each other, a molecular feature reflecting their biological function in the infectious process. The effects of artificial mutation accumulation in each of eIF4E and VPg proteins encoding genes was assessed by monitoring VPg-eIF4E interaction using yeast two hybrid (Y2H) system. More specifically, independent screenings of either VPg or eIF4E mutant libraries for the interaction with their respective wild type partner were performed.

## Materials and methods

In order not to lose the high quality (size and diversity) of the libraries during their transfer from bacteria to yeasts, a cloning strategy was optimized. We used high efficiency Gateway recombination system, bacteria strains with maximum transformation efficiency and yeast mating that typically insures a better rate of cotransformed yeast. Corresponding mating efficiencies in yeast are provided in S2 Table. Typically, Libraries transformation in *E*.*Coli* produced an average of 3x10^4^ individual clones. The higher cloning-mating efficiency in yeast *(*>10^6^) insured that the original library complexity was conserved during the transfer between bacteria and yeast (see supporting information for details).

### Gene sequences used in the study

Viral genome linked protein (VPg) was from a variant of PVY isolate SON41 (GenBank accession AJ 439544–2)[25]. The Eukaryotic translation initiation factor 4E used in this study is encoded by *Capsicum annuum* Yolo Wonder, a bell pepper inbred line susceptible to PVY (GenBank accession AAN74644-1).

### Production of random mutant libraries

Genes encoding Viral protein genome-linked protein (VPg) from Potato virus Y (PVY) SON41g and eIF4E factor from *Capsicum annuum* cv. Yolo Wonder were cloned into pDONR201 entry vector Gateway system (ThermoFisher). GeneMorph II Random Mutagenesis Kit (Agilent) was used to perform random error-prone PCR (epPCR) mutagenesis. Several DNA target amounts were tested to produce various rates of mutations (S3 Table). In case of "Highly-mutated" libraries, an iterative strategy was used, involving two successive rounds of PCR-mutagenesis. EpPCRs were performed following manufacturer instructions and using attL1 and attL2 primers (5'TCGCGTTAACGCTAGCATGGATCTC and 5'GTAACATCAGAGATTTTGAGACAC respectively) that hybridize upstream and downstream of attL Gateway recombination sites. PCR products were loaded on agarose gel 1%. Amplicons of the expected size were extracted from the gel using NucleoSpin Gel and PCR Clean-up kit (Macherey-Nagel). AttL-flanked mutated eIF4E and VPg genes were inserted into Yeast Two Hybrid pDEST-GADT7 (activation domain fusion vector—TAIR resource accession #1010229695) and pDEST-GBKT7 (DNA-binding domain fusion vector—TAIR resource accession #1010229688) respectively. The eIF4E auto-activation prevented us from using the "eIF4E-binding domain" construct. LR recombination reactions were performed at 25°C overnight using LR Gateway LR Clonase II Enzyme mix following manufacturer recommendations. Proteinase K treatment were applied by adding 1μl of proteinase K and incubation at 37°C for 1 minute. LR products were used to transform *Escherichia coli* DH10B MAX-efficiency competent cells (NEB) by electroporation. Transformation products were spread and transformed cells selected on LB plates supplemented with the suitable antibiotics (Kanamycin or Ampicillin). To characterize the libraries, 32 colonies per library were sequenced using the attB1 primer (Gateway). Each resulting sequences were aligned with the WT sequence of the corresponding gene to identify mutations. Total number of mutations, number of non-synonymous, synonymous, STOP and INDEL (insertion-deletion) mutations and their respective position in the protein sequence are displayed in S1 Fig. Standard Kruskal-Wallis statistical tests were performed to estimate the homogeneity of the number of non-synonymous mutations per clone between eIF4E and VPg of a given library (low, medium, high). Statistical homogeneity of INDEL and STOP mutations proportion between eIF4E and VPg libraries were checked using Z-score calculation. Theoretical frequencies of STOP mutations and non-synonymous ones were assessed from PEDEL-AA analysis web-tool [26]. For each mutant library, all isolated colonies were retrieved from LB plates and suspended into liquid LB media for 6 hours. Plasmid extractions were performed using Nucleospin Plasmid kit (Macherey-Nagel). DNA mutant plasmid libraries were suspended in water and stored at -20°C.

### Yeast-Two-Hybrid analysis

The Matchmaker GAL4 Two-Hybrid system 3 (Clontech) was used according to the manufacturer specifications. pDEST GADT7—containing either the wt eIF4E sequence or the different mutant libraries and pDEST GBKT7- containing either the wt VPg sequence or the different mutant libraries were transformed into AH109 and Y187 yeast strains respectively, which contain two independent reporter genes HIS3 and ADE2, conferring histidine and adenine auxotrophy, respectively and driven by hybrid GAL4 promoters. Two-hybrid library screening using yeast mating were performed following the recommendations of the MatchmakerTM prefransformed libraries user manual (Clontech PT 3183–1) and the Make your own “Mate and PlateTM” library system user manual (Clontech PT 4085–1). Each of the yeast mutant libraries “low”, “medium”, “high” were screened using yeast mating with the wild type partner: 5 ml of the wt interacting partner strain (>10^8^ cells/ml) was combined with 1ml aliquot of the mutant library strain (> 10^7^ cells/ml). Diploid yeasts were selected on a non-interaction selective medium (without leucine and tryptophan, -LW), the number of colonies represented the whole population of variants. Yeast colonies able to grow on stringent selecting medium for interaction (without leucine, tryptophan, histidine and adenine, -LWHA) representing the remaining functional variants were counted on 3 to 5 independent plates. For each experiment, mating efficiencies i.e., % diploid) calculated as the percentages of cfu/ml of diploids (number of cfu on–LW plates) related to cfu/ml of the lower viability partner, was > 7%. The number of clones screened, calculated as the number of cfu/ml of diploids x resuspension volume, was estimated for each mating and was > 10^6^. Yeast colonies were counted both manually and with Open CFU, an open-source software, http://opencfu.sourceforge.net, [27]

## Results

### VPg and eIF4E randomized mutant libraries characterization

In this work, we postulated that the mutational robustness of VPg and eIF4E could be compared by monitoring the loss of VPg-eIF4E interaction as a function of a progressive accumulation of random mutations into each of the two proteins.

**Fig 1:**
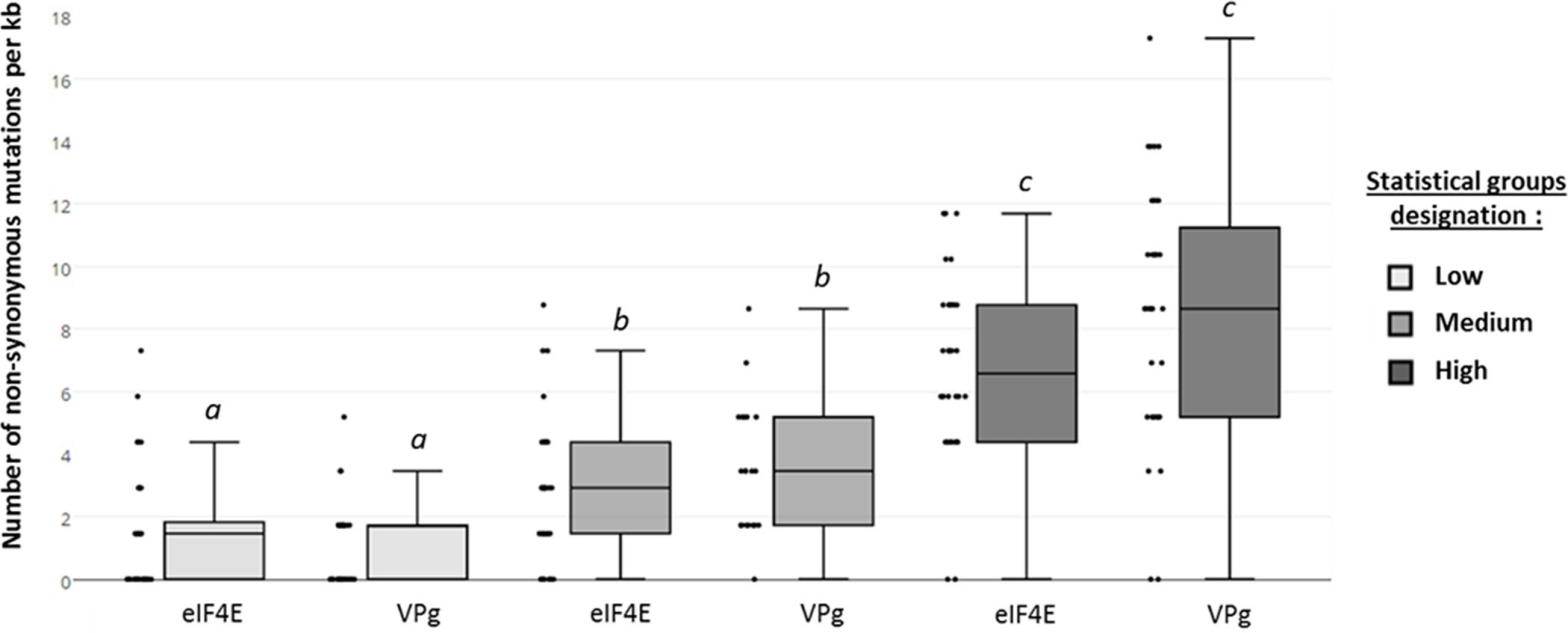
Distribution of the number of non-synonymous mutations in sequenced variants sampled from each VPg and eIF4E mutant library. Black dots: number of NS mutation per kb retrieved from each of the 32 variants sequenced. *a*, *b* and *c* are 3 significantly different statistical groups (p-value cut-off at 0.01, Kruskal-Wallis test).

To introduce molecular diversity into parent sequences of the viral protein VPg and its cellular partner eIF4E, three libraries of randomized VPg and eIF4E mutants were generated using error-prone polymerase chain reaction (epPCR). The mutational rate was modulated by finely tune the amount of matrix. For high mutational rates, two consecutive epPCR amplifications of the starting material were performed (see experimental section). Pools of 32 individual clones were randomly chosen in each library and sequenced. Non synonymous mutations leading to stop codons or frame shifts were not considered. Taking into account nucleotide mutations leading to amino acid changes (non-synonymous mutations, NS), the libraries were classified as "low", sequences containing an average of 1.5 NS mutations per kilo base (kb), “medium” (3 NS mutations) or “high” (7 NS mutations) (Fig 1).

For both eIF4E and VPg coding sequences, the “low”, “medium” and “high” statistical groups were significantly different from each other (p-value between 0.0001 and 0.01, Kruskall-wallis test).

To accurately compare the mutational robustness of the two proteins, the mutation rate of eIF4E and VPg variant libraries within each of the three statistical groups has to be not significantly different (p-value >0.01, Table 1).

**Table 1:** Comparison of the 6 libraries used in this study.

Mutants libraries statistical comparison		High		Medium		Low	
		4E YW	VPg S41g	4E YW	VPg S41g	4E YW	VPg S41g
**High**	4E YW	x	***n*.*s***	***	***	***	***
	VPg S41g		x	***	***	***	***
**Medium**	4E YW			x	***n*.*s***	*	*
	VPg S41g				x	**	**
**Low**	4E YW					x	***n*.*s***
	VPg S41g						x

Side by side comparisons of average non-synonymous mutation number per variant in eIF4E and VPg mutant libraries. "***": p-value< 0.0001

"**": p-value< 0.001

"*": p-value< 0.01; “n.s”,not significant. For p-value> 0.01, Kruskal-Wallis test, libraries were considered as non-significantly different.

**Fig 2:**
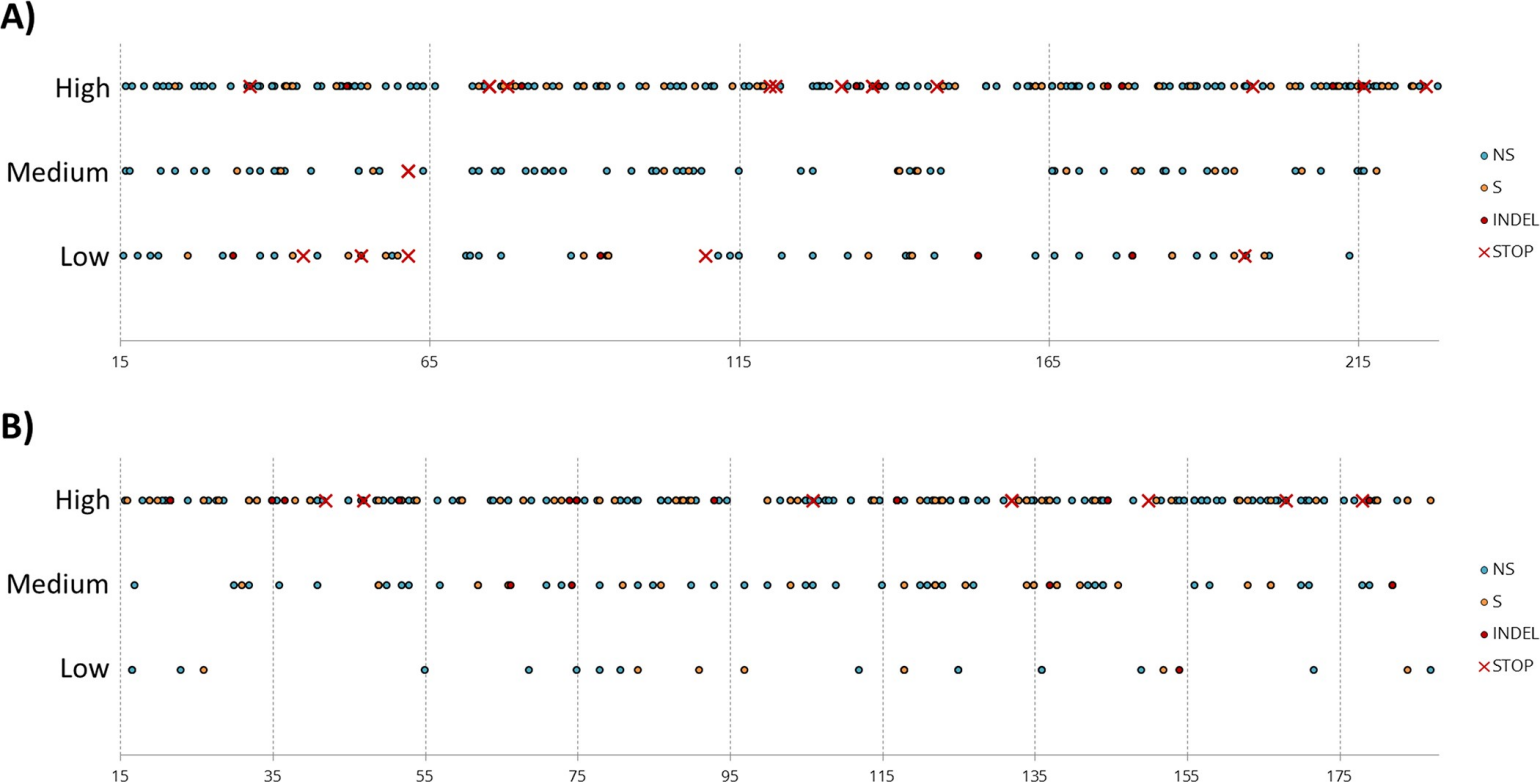
Distribution along eIF4E and VPg sequences of all mutations found in the 32 randomly chosen variants sequenced in each of the six libraries. **A)** eIF4E; **B)** VPg. NS: non-synonymous; S: synonymous; INDEL: insertion/deletion; STOP: mutation involving the appearance of STOP-codons. For comparative purpose, number of mutations is displayed rather than percentages of mutated sites in the coding sequence, as the two proteins are of comparable length.

The mutagenesis coverage was estimated by locating on the coding sequence all the mutations observed in each of the sequenced samples. For each of the six libraries within a given statistical class (VPg and eIF4E “low”, “medium” and “high” libraries), mutations were evenly distributed with a homogenous coverage along the gene sequence (Fig 2).

### Analysis of library representativeness

Good library representativeness must satisfy a high diversity (i.e. covering a significant number of possible mutations). This corresponds to a library containing as few redundant sequences (including copies of the non-mutated parental gene) and as many full-length sequences (lacking premature termination codons) as possible. Bacteria were transformed with the six libraries and, from positive clones counting it was deduced that the libraries contained an average of 3.5 10^4^ clones. Sequencing results and their derived nucleotide substitution matrixes (S2 Fig) were used to feed the PEDEL-AA web-tool [26]. Starting from the overall (DNA) mutation rate, the total size of the library and the nucleotide mutation matrix (which was obtained here by sequencing randomly chosen library members) PEDEL-AA estimates the protein sequence diversity in error prone PCR libraries. Notably, the most useful feature of PEDEL-AA is its ability to quickly estimate the total number of unique proteins in an epPCR library. Using this algorithm, the protein diversity obtained from random mutagenesis of eIF4E and VPg at identical mutation rates was derived (S1 Table). According to this analysis, eIF4E and VPg-based random mutagenesis were expected to provide very similar STOP-mutation frequencies (differences representing less than 10%) in accordance with sequencing results on the 32 randomly chosen variants. NS vs. Synonymous (S) mutation ratios obtained from eIF4E and VPg theoretical libraries revealed minor differences. The VPg “medium” and “high” libraries were expected to provide slightly more non-synonymous mutations (excluding stop codon) per kb than eIF4E. According to this analysis, if a different mutational robustness is observed for the two proteins, it should more likely refer to different non-synonymous mutation tolerance rather than to a differential STOP-codon or INDEL accumulation.

### Comparative analysis of VPg and eIF4E mutational robustness

**Fig 3:**
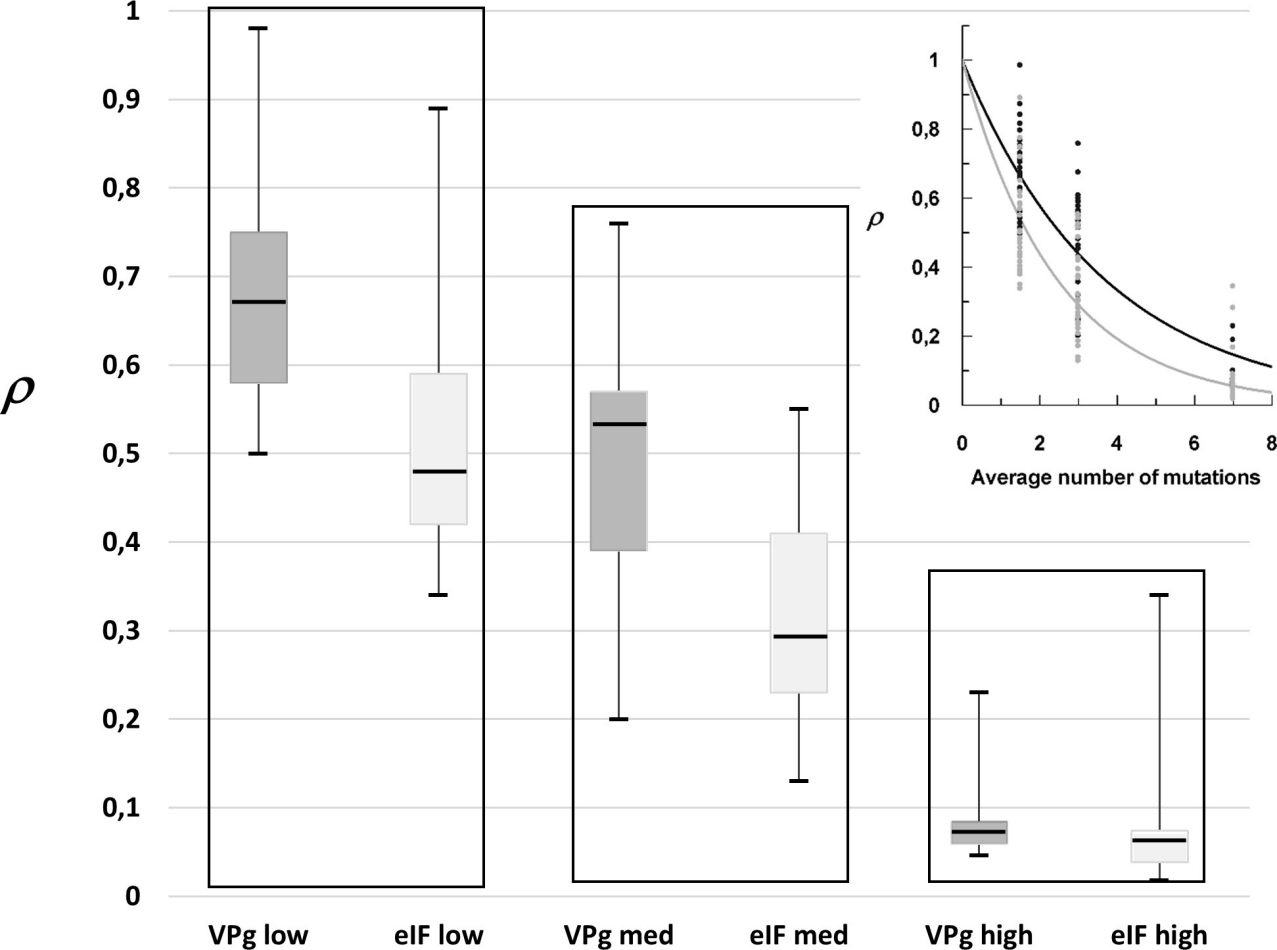
Comparative mutational robustness of VPg (dark grey) and eIF4E (light gray). Box plots display of the dispersion of remaining functional variants of VPg (dark grey) and eIF4 (light gray) within the low, med and high libraries. Yeasts transformed with “low”, “medium” and “high” libraries of variants were matted with yeasts transformed with the wild type partner encoding gene. The resulting diploid yeasts containing the genes of both protein partners were selected on a non-interaction selective medium (-LW). The corresponding number of colonies, ***τ***, represented the whole population of variants. Yeast colonies able to grow on stringent interaction selecting medium (-LWHA) were counted on 3 to 5 independent plates (Three independent panning). The corresponding number, ***σ***, was divided by ***τ***. ***ρ*** = ***σ*** /***τ*** refers to the rate of remaining functional variants within the mutants libraries relative to the starting whole population of variants. ***ρ*** is indicative of the mutational robustness of the protein considered. (See Material and Methods for more details). (t-test p-value <0.01). Inset: the rate of remaining functional variants versus mutation accumulation (black circles and gray circles, VPg and eIF4E library survival rates respectively). The mono exponential model of fitness decline as a function of mutation accumulation was fitted to the experimental data ***ρ*** = EXP(-k*n) with n, the averaged mutation number per protein. Best fit: *k* = 0.274±0.011 (VPg black line) and *k* = 0.412±0.016 (eIF4E gray line).

Comparing the mutational robustness of two proteins of unrelated functions does not allow to put the results obtained in a true biological perspective. By contrast the choice of comparing VPg and eIF4E mutational robustness through the evaluation of mutation accumulation effects on their interaction is especially relevant. Indeed, the VPg-eIF4E interaction is required for potyvirus infection. More specifically, with respect to evolutive aspects, mutations in eIF4E are related to resistance mechanisms to the virus, which are in turn overcome by virus adaptation through mutations within the intrinsically disordered VPg central region [28]. Among the numerous VPg natural variants and eIF4E alleles provided by the PVY-pepper pathosystem, Pvr^2+^-eIF4E allele and VPg from PVY SON41g isolate were chosen, as Y2H test reveals their strong interaction, thereby reducing the occurrence of false positives (Charron et al. 2008). In order to compare the mutational robustness of both proteins, Y2H experiments were performed by parallel screening of eIF4E mutated libraries against wild type VPg and reversely, mutated VPg libraries against wild type eIF4E. For each of the three groups of libraries (low, medium and high), the VPg and eIF4E mutant populations were compared across all mutagenesis conditions (t-test p-value <0.01, Fig 3) and also within each mutagenesis condition (t-test for low, medium and high libraries, p-values <0.0002, <0.001 and <0.5 respectively). Although no significant difference was observed between the two proteins at high mutation rate, taken together the data show that the remaining functional populations of VPg variants was significantly higher than the corresponding population of eIF4E variants (Fig 3). Such discrepancy could be explained by differences in codon volatility, a property linked to the nucleotide sequence encoding each of the two proteins. Volatility of a codon is defined as the probability that a random point mutation in this codon is non synonymous [29]. If the nucleotide sequence encoding VPg displays a significantly lower codon volatility than the sequence encoding eIF4E, a given mutation rate could generate more synonymous codons in the VPg sequence, ensuing into more wild type proteins within the population, and in turn would not imply a higher mutational robustness at the level of the VPg amino acid sequence. There is no bias of this kind, as according to our calculation, VPg and eIF4E nucleotide sequences display comparable probabilities of synonymous codon apparition (0.3) upon random point mutations. An apparently higher mutation robustness of VPg compared to eIF4E can be observed from 1.5 to 7 non-synonymous mutations per kb.

## Discussion

### The intrinsically disordered VPg displays a higher mutational robustness than eIF4E, its more structured partner

Several factors could explain the apparently higher mutation robustness observed in VPg compared to eIF4E. Firstly, this difference could be a general property of the source organism (i. e. virus vs. eukaryotic) rather than specific properties of the proteins involved. This seems unlikely, as in eukaryotic proteomes, IDRs have a higher amino acid polymorphism than ordered regions [30], a feature also observed in virus proteomes. This is well illustrated in potyviruses, where IDRs tend to evolve faster than more structured regions, [11]. Another explanation could be that each amino-acid site of eIF4E and VPg are equally tolerant to mutations, but that a higher proportion of sites in eIF4E are involved in the interaction. This could mean that the distribution over the protein structure, of amino acids involved in the interaction is wider for eIF4E than for VPg. However, the interaction domains, namely the central region of the VPg [31–33] and the C-terminal region of eIF4E [21] are of the same order (20–30 residues length). Hence, it is likely that the VPg structural elements which are involved in the interaction, better tolerate the accumulation of non-synonymous mutations than their eIF4E counterparts. By contrast, the loss of eIF4E interaction properties illustrates the stronger structural constraints exerted on this factor.

It was previously deduced from hydrodynamic parameters determination that VPg behaves as a pre-molten globule [34]. This type of protein possesses loosely packed cores involving secondary structures interspaced with disordered regions [35]. Viral proteins display a high occurrence of such features. A comparative thermodynamic analysis between compact proteins from thermophilic organisms and viral proteins was previously reported. Although the set of structures available was limited, the study strongly suggested that intrinsically disordered viral proteins and compact proteins could display two different types of functional loss, with respect to mutation accumulation [13]. If deleterious mutations interact so that their combined impact on function is greater than expected from an addition of their individual effect, the function decrease would accelerate with the accumulation of mutations, giving rise to negative epistasis. Compact stable globular proteins would comply to this model, namely “threshold robustness”, whereas, many viral proteins would obey the “gradient robustness” model, with a lower average loss per mutation [15]. This hypothesis paved the way to the present work. The impact of mutations depends both on the effect of each mutation on the formation of VPg-eIF4E complex and on the interaction between accumulated mutations. We could not tune the random mutagenesis parameters finely enough such as being able to generate additional mutant libraries in between our three non-overlapping libraries (Fig 1). Consequently, the shape of the curve of ***ρ*** versus the average mutation number could not be more precisely defined in order to explore a possible negative epistasis. Hence, we simply hypothesized that deleterious mutations were not interacting (no epistasis). In such a case, the VPg-eIF4E interaction, expressed as the survival rate, was expected to decline mono-exponentially. It was observed that the functional loss of the two proteins decreased with significantly different rates, which is indicative of a higher mutational robustness of VPg than eIF4E, its more structured partner (Fig 3).

Previous extensive computational analyses of disordered proteins and their evolution [36,37] converge to the agreement that disordered protein regions evolve rapidly. We recently analyzed both intra and interspecies amino acid polymorphism within the potyvirus proteome. Regarding the VPg, there are three intrinsically disordered regions spanning the first fifth of the N-terminal part, the last fifth of the C-terminal part and the central part, roughly between amino-acid 85 and 120. The latter displays a larger amino acid polymorphism than the other parts of the protein [11]. This region, which is involved in the interaction with eIF4E, is under positive selection with respect to amino acids determining the host resistance breakdown [38]. As a matter of fact, large phylogenetic analysis shows a significantly higher degree of positive Darwinian selection in intrinsically disordered regions compared to more structured regions. Such a positive selection is significantly enriched in classes of intrinsically disordered proteins whose functions can be coupled to adaptive processes [39].

IDRs are known to adopt different conformations upon interaction with different binding partners [40]. Interestingly, interactions with multiple partners and high rates of evolution are both associated with disorder [41,42] and conformational flexibility [43]. It was shown that the intrinsically disordered central region of VPg participates in the interaction with the virus helper component HC-Pro, eIF4E being a competitive binder of this latter [33]. It is likely that intrinsic disorder brings the conformational flexibility required for this multipartnership. Indeed, binding promiscuity is one of the functional features of intrinsically disordered regions [44,45]. Although it seems counterintuitive that a region participating to the definition of a precise surface complementation in the context of a protein-protein interaction displays mutational robustness, it was observed that there are several possibilities to bind a given protein. The surface overlap may be defined by alternative combinations of amino acids [46].

### Mutational robustness in VPg relates to the particular class of “flexible disorder”

We previously observed that, globally, in potyviruses, IDRs display significantly higher dN/dS (non-synonymous substitutions over synonymous substitutions) values than ordered ones, that indicates a tendency of intrinsically disordered domains to evolve faster than more structured regions during potyvirus evolution [11]. Such weaker evolutive constraints on IDRs strongly suggest abilities for a faster and easier exploration of adaptive solutions and the emergence of new functions in viral proteins [13,47]. However, disorder is not systematically related to mutational robustness. Bellay and colleagues proposed to sort conserved disordered regions in two classes according to their evolutive rates: “flexible disorder”for disordered regions which display a relatively high amino acid polymorphism, and “constrained” disorder which refers to disordered regions that are not variable in their amino acid sequences [48]. Flexible disorder is, in the first instance, found in flexible spacers between functional domains [49]. It is also observed in proteins with one or two regions interacting sequentially with several partners through conformational switches, termed “date Hubs” [50]. VPg belongs to this family, as its intrinsically disordered central region sequentially interacts with eIF4E and the virus helper component HC-Pro [33]. In addition VPg interacts also with CI, the viral helicase [51]. During the preparation of this manuscript, the involvement of VPg N-ter disordered region in an interaction with NIa-Pro, the protease domain responsible for the viral polyprotein maturation was reported. NIa-Pro protease activity and VPg ATPase activity are modulated through this binary interaction [52]. This finding supports the hypothesis that VPg belongs to the family of conformational switches, a family of IDPs displaying mutational robustness. By contrast, constrained disorder is found in IDRs, whose function is linked to more strict topological requirements. This is the case of multi-interface hubs (named “party hubs”) [50] that can be involved in simultaneous binding with various partners.

### Conclusion

The experimental analysis of intrinsically disordered protein mutational robustness is still scarcely documented. Recently, the demonstration that the C-terminal IDR of Nodamura Virus polymerase can accommodate very diverse amino acid sequences without losing its function suggests that IDRs can be reservoirs for evolutionary innovations towards virus adaptation to environment changes [14]. This hypothesis needs to be assessed using other examples. The strength of our experimental model lies in the fact that it makes it possible to compare the mutational robustness of two naturally interacting proteins in a biological context. The results of the present study supports previous conclusions. Indeed, using the same experimental model, we recently observed *in planta* that a modulation of the VPg disorder content impacts the success of the virus to overcome the host resistance [12]. The questions raised by the possible link between IDR mutational robustness and adaptive processes is of very general relevance. This type of study requires to be further developed as it will feed the debate regarding the current reassessment of the dogma of the relationship between protein structure and function. To this end, reverse genetics is especially suitable. Using phytovirus as experimental models, the biological properties of mutants can be quickly and accurately tested *in vivo* on large populations of host plants (1000 and more). Notably, a comparative analysis in *planta* of the impact on infection phenotype of mutations introduced either in ordered or disordered regions within the virus genome will allow the assessment of IDRs mutational robustness in a true biological context and ultimately, to better understand how viruses cope with mutations.

## Supporting information

S1 Fig Mean substitution rates matrixes. (PDF)

S2 Fig Distribution of VPg and eIF4E codon volatility. (PDF)

S1 Table Library characteristics (PEDEL-AA analysis). (PDF)

S2 Table Mating efficiency in yeast. (PDF)

S3 Table Error prone PCR conditions used to produce “low”, “medium” and “high” libraries. (PDF)

S4 Table Mutations within the six (low, med, high) samples sequenced to characterize VPg and eIF4 mutant libraries. (PDF)

S1 Code Rcode for codon volatility calculation. (PDF)
