# Supplementary material for: Comparative analysis of mutational robustness of the intrinsically disordered viral protein VPg and of its interactor eIF4E
Source: PLoS One. 2019 Feb 14;14(2):e0211725. doi: 10.1371/journal.pone.0211725 (PMC6375565; doi:10.1371/journal.pone.0211725)
Supplement: S1 Fig — (PDF) [file pone.0211725.s001.pdf]

Comparative analysis of mutational robustness of intrinsically disordered viral protein VPg and of its interactor eIF4E

Jocelyne Walter, Justine Charon, Yihua Hu, Joy Lachat, Thomas Leger, Guillaume Lafforgue, Amandine Barra, Thierry Michon

SUPPORTING INFORMATION

S1 Fig. Mean substitution rates matrixes
S2 Fig. Distribution of VPg and eIF4E codon volatility
S1 Table. Library characteristics (PEDEL-AA analysis)
S2 Table. Matting efficiency in yeast
S3 Table. Error prone PCR conditions
R script for codon volatility calculation
S4 Table. Mutations within the six (low, med, high) samples sequenced to characterize the VPg and eIF4 mutant libraries.

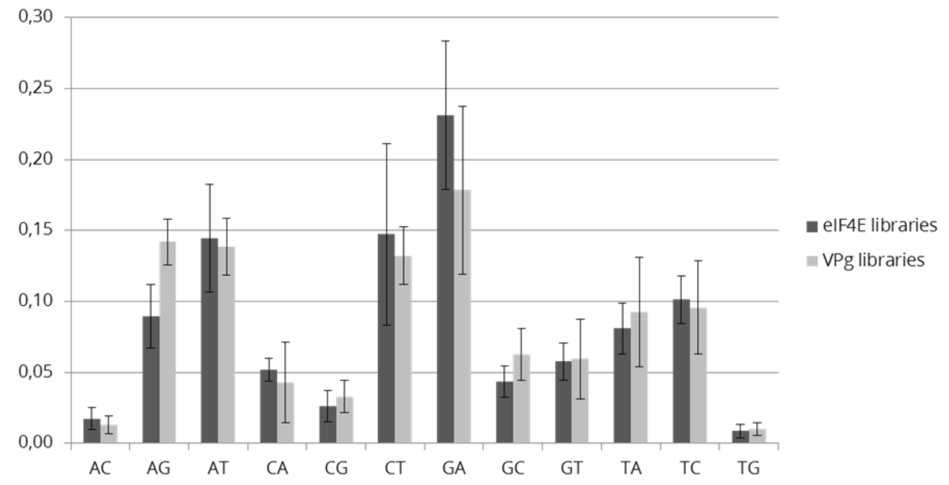

S1 Fig. Mean substitution rates obtained for eIF4E and VPg libraries. Vertical bars represent standard errors.
