# Supplementary material for: Comparative analysis of mutational robustness of the intrinsically disordered viral protein VPg and of its interactor eIF4E
Source: PLoS One. 2019 Feb 14;14(2):e0211725. doi: 10.1371/journal.pone.0211725 (PMC6375565; doi:10.1371/journal.pone.0211725)
Supplement: S2 Fig — (PDF) [file pone.0211725.s002.pdf]

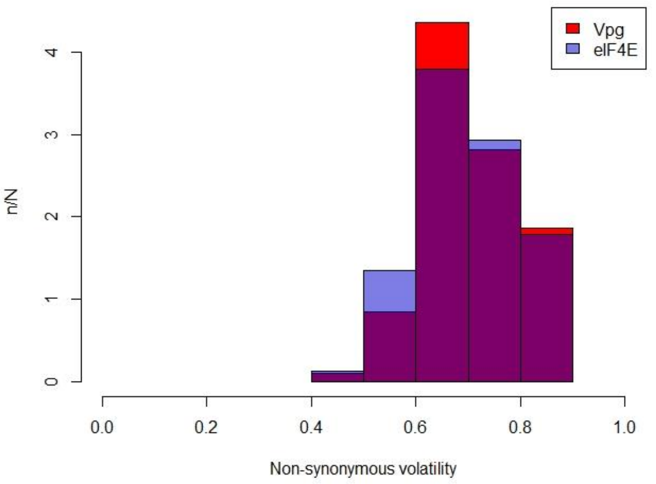

S2 Fig. Distribution of VPg and eIF4E codon volatility. n/N is defined as codon volatility density, with n : number of codon within a range of volatility values and N : total number of codons for a given protein sequence.
