# Supplementary material for: Comparative analysis of mutational robustness of the intrinsically disordered viral protein VPg and of its interactor eIF4E
Source: PLoS One. 2019 Feb 14;14(2):e0211725. doi: 10.1371/journal.pone.0211725 (PMC6375565; doi:10.1371/journal.pone.0211725)
Supplement: S1 Table — (PDF) [file pone.0211725.s003.pdf]

S1 Table. Library characteristics (PEDEL-AA analysis). The size of each library was determined. For each of the 6 libraries, 32 variants were randomly chosen and sequenced. From these data, a nucleotide substitution matrix (figure S2) was derived together with the mean number of nucleotide substitutions, insertions and deletions (INDEL). These elements were used to feed the PEDEL-AA algorithm, which returned the specifications expected for each of the 6 libraries.

Total library size : 3 10^4

	eIF4E low	VPg low	eIF4E med	VPg med	eIF4E high	VPg high
Number of distinct full-length proteins in the library	9543	4682	13790	14160	14200	14500
Unmutated (wild-type) sequences (% of library)	23.30	33.40	16.10	7.47	0.62	0.21
mean number of AA substitutions per variant (PEDEL-AA)	1.23	0.76	1.66	2.20	4.43	5.46
mean number of AA substitutions per variant (experimental)	1.16	0.47	1.94	2.05	4.56	5.37
number of variants with no INDEL or stop codons	23.9×10^3	21.4×10^3	25.4×10^3	20.5×10^3	14.6×10^3	14.6×10^3
