# Supplementary material for: Comparative analysis of mutational robustness of the intrinsically disordered viral protein VPg and of its interactor eIF4E
Source: PLoS One. 2019 Feb 14;14(2):e0211725. doi: 10.1371/journal.pone.0211725 (PMC6375565; doi:10.1371/journal.pone.0211725)
Supplement: S2 Table — (PDF) [file pone.0211725.s004.pdf]

S2 Table. Matting efficiency in yeast. Mating efficiency (percentage of diploids) was calculated as : number of cfu/ml of diploids/ number of cfu/ml of limiting partner x 100 =% of diploids. Matting efficiencies (two independent series) were all in between 15% and 33%. Haploïd yeast cells carrying eIF4E and VPg libraries (average of 10^8 cells/mL) were mated with haploïd yeast cells bearing the wt VPg or eIF4E gene. According to matting efficiency, at least $15x10^6$ diploïd cells containing both genes were obtained for interaction screening. This insured that the diversity of the $3x10^4$clones transferred from *E. coli* to yeast was preserved.

Serie 1

Name	VPg WT	VPg Low	VPg Medium	VPg High
**eIF4E WT**	15% ; 24%	21%	26%	16%
**eIF4E low**	26%			
**eIF4E Med**	27%			
**eIF4E High**	30%			

Serie 2

Name	VPg WT	VPg Low	VPg Medium	VPg High
**eIF4E WT**	20%	19%	18%	17%
**eIF4E low**	33%			
**eIF4E Med**	25%			
**eIF4E High**	30%			
