# Supplementary material for: Comparative analysis of mutational robustness of the intrinsically disordered viral protein VPg and of its interactor eIF4E
Source: PLoS One. 2019 Feb 14;14(2):e0211725. doi: 10.1371/journal.pone.0211725 (PMC6375565; doi:10.1371/journal.pone.0211725)
Supplement: S3 Table — (PDF) [file pone.0211725.s005.pdf]

S3 Table. Error prone PCR conditions used to produce "low", "medium" and "high" libraries. Amount of DNA target used to generate eIF4E and VPg mutant libraries, using ep-PCR random mutagenesis. 30 cycles amplifications were performed . pDONR201-eIF4E YW and pDONR201-VPg SON41g were used as DNA templates to obtain eIF4E and VPg mutant libraries, respectively . For generating VPg-high and eIF4E-high, the highly mutated libraries, two successive epPCR were performed, (int) stands for conditions of the first epPCR round.

Library name	Amount of target gene	Total Amount of plasmid
eIF4E-Low	720ng	2200ng
VPg-Low	600ng	2000ng
eIF4E-Medium	45ng	137ng
VPg-Medium	64ng	200ng
eIF4E-High<br>*eIF4E-(int)High*	15ng from eIF4E-(int)High<br>*14ng*	-<br>*42ng*
VPg-High<br>*VPg-(int)High*	6ng from VPg(int)High<br>*6ng*	-<br>*20ng*
