# Supplementary material for: Comparative analysis of mutational robustness of the intrinsically disordered viral protein VPg and of its interactor eIF4E
Source: PLoS One. 2019 Feb 14;14(2):e0211725. doi: 10.1371/journal.pone.0211725 (PMC6375565; doi:10.1371/journal.pone.0211725)
Supplement: S4 Table — (PDF) [file pone.0211725.s006.pdf]

S4 Table. Mutations within the six (low, med, high) samples sequenced to characterize the VPg and eIF4 mutant libraries.

Library sample	eIF4E LOW									
**total mutations**	**60**		**4**	**32**	**26**	**37**	**14**	**5**		0
**mutant ref.**	**Mutations**	**nt Position**	**INDEL**	**Transition**	**Transversion**	**NS**	**S**	**STOP**	**aa Substitution**	**aaPosition**
**430**	**1**		**0**	**0**	**1**	**0**	**1**	**0**	**1**	**0**
	AT	162		0	1		1		PP	54
**431**	**5**		**0**	**2**	**3**	**4**	**0**	**1**	**5**	**0**
	GT	60		0	1	1			LF	20
	GA	230		1	0	1			SN	77
	AT	341		0	1	1			KM	114
	GT	575		0	1	1			SI	192
	GA	590		1	0			1	**STOP**	197
**432**	**4**		**0**	**1**	**3**	**2**	**1**	**1**	**4**	**0**
	CA	113		0	1	1			TN	38
	TA	329		0	1			1	**STOP**	110
	AG	440		1	0	1			YC	147
	AC	555		0	1		1		AA	185
**433**	**1**		**1**	**0**	**0**	**0**	**0**	**0**	**1**	**0**
	INSERTION	536	1	0	0					179
**434**	**2**		**0**	**0**	**2**	**1**	**1**	**0**	**2**	**0**
	TA	180		0	1		1		TT	60
	GC	379		0	1	1			AP	127,0
**435**	**1**		**0**	**1**	**0**	**0**	**1**	**0**	**1**	**0,0**
	GA	129		1	0		1		SS	43
**436**	**4**		**0**	**2**	**2**	**3**	**0**	**1**	**4**	**0,0**
	TA	120		0	1	1			DE	40
	TA	134		0	1			1	**STOP**	45

	TC	175		1	0	1			WR	59,0
	GA	553		1	0	1			AT	185,0
**437**	**7**		**0**	**5**	**2**	**5**	**2**	**0**	**7**	**0,0**
	AG	47		1	0	1			EG	16
	AT	366		0	1	1			ED	122
	AT	398		0	1	1			KI	133
	CT	429		1	0		1		TT	143
	AG	487		1	0	1			IV	163,0
	GA	496		1	0	1			AT	166,0
	TC	600		1	0		1		FF	200
**439**	**2**		**1**	**1**	**0**	**1**	**0**	**0**	**2**	**0,0**
	INSERTION	100	1	0	0					33
	CT	428		1	0	1			TI	143
**406**	**1**		**0**	**1**	**0**	**1**	**0**	**0**	**1**	**0,0**
	TC	536		1	0	1			LS	179
**407**	**4**		**1**	**2**	**1**	**3**	**0**	**0**	**4**	**0,0**
	GA	95		1	0	1			GD	32
	GA	217		1	0	1			AT	73,0
	INSERTION	461	1	0	0					154
	GT	591		0	1	1			WC	197
**408**	**0**		**0**	**0**	**1**	**0**	**0**	**0**	**0**	**0**
				0	1					0
**409**	**0**		**0**	**0**	**1**	**0**	**0**	**0**	**0**	**0**
				0	1					0
**410**	**1**		**0**	**1**	**0**	**0**	**1**	**0**	**1**	**0**
	GA	585		1	0		1		KK	195
**15**	**0**		**0**	**0**	**0**	**0**	**0**	**0**	**0**	**0**
										0
**16**	**1**		**0**	**1**	**0**	**0**	**1**	**0**	**1**	**0**
	TC	270		1	0		1		DD	90

**17**	**2**		**0**	**2**	**0**	**1**	**1**	**0**	**2**	**0**
	AG	215		1	0	1			QR	72
	TC	282		1	0		1		AA	94
**18**	**3**		**1**	**0**	**2**	**2**	**0**	**0**	**3**	**0**
	INSERTION	278	1	0	0					93
	GC	335		0	1	1			CS	112
	GC	424		0	1	1			DH	142,0
**19**	**1**		**0**	**0**	**1**	**0**	**0**	**1**	**1**	**0**
	AT	526		0	1			1	**STOP**	176,0
**19BIS**	**0**		**0**	**0**	**0**	**0**	**0**	**0**	**0**	**0**
										0
**20**	**2**		**0**	**2**	**0**	**1**	**0**	**1**	**2**	**0**
	GA	185		1	0			1	**STOP**	62
	CT	343		1	0	1			HY	115,0
**21**	**0**		**0**	**0**	**0**	**0**	**0**	**0**	**0**	**0**
										0
**22**	**0**		**0**	**0**	**0**	**0**	**0**	**0**	**0**	**0**
										0
**23**	**1**		**0**	**1**	**0**	**0**	**1**	**0**	**1**	**0**
	TC	408		1	0		1		FF	136
**24**	**1**		**0**	**1**	**0**	**1**	**0**	**0**	**1**	**0**
	GA	52		1	0	1			VM	18,0
**25**	**1**		**0**	**1**	**0**	**1**	**0**	**0**	**1**	**0**
	GA	508		1	0	1			VI	170,0
**26**	**2**		**0**	**2**	**0**	**2**	**0**	**0**	**2**	**0**
	GA	64		1	0	1			AT	21,3
	GA	262		1	0	1			VI	88,0
**27**	**1**		**0**	**1**	**0**	**0**	**1**	**0**	**1**	**0**
	TC	78		1	0		1		DD	26
**28**	**2**		**0**	**2**	**0**	**1**	**1**	**0**	**2**	**0**

	AG	215		1	0	1			QR	72
	TC	282		1	0		1		AA	94
**29**	**0**		**0**	**0**	**0**	**0**	**0**	**0**	**0**	**0**
										0
**30**	**5**		**0**	**2**	**3**	**4**	**1**	**0**	**5**	**0**
	AT	141		0	1	1			KN	47
	GA	156		1	0		1		KK	52
	AT	213		0	1	1			KN	71
	CT	281		1	0	1			AV	94
	CA	343		0	1	1			HN	115,0
**31**	**2**		**0**	**0**	**2**	**1**	**1**	**0**	**2**	**0**
	AT	174		0	1		1		SS	58
	TA	602		0	1	1			LQ	201
**32**	**3**		**0**	**1**	**2**	**3**	**0**	**0**	**3**	**0**
	AC	528		0	1	1			KN	176
	GC	565		0	1	1			AP	189,0
	AG	641		1	0	1			DG	214

TA	5
AT	8
CG	0
GC	4
TG	0
GT	3
GA	14
AG	6
CA	2
AC	2
TC	8
CT	4

INSERTION 4
DELETION 0

**Library sample**	**VPg LOW**									
**total mutations**	**25**	**0**	**1**	**15**	**10**	**16**	**8**	**0**		0
**Mutant ref**	**Mutations**	**nt Position**	**INDEL**	**Transition**	**Transversion**	**NS**	**S**	**STOP**	**aa substitution**	**aa Position**
**1**	**0**		**0**	**0**	**0**	**0**	**0**	**0**	**0**	**0**
										0
**2**	**1**		**0**	**1**	**0**	**0**	**1**	**0**	**1**	**0**
	GA	354		1	0		1		LL	118
**3**	**0**		**0**	**0**	**0**	**0**	**0**	**0**	**0**	**0**
										0
**4**	**2**		**0**	**2**	**0**	**1**	**1**	**0**	**2**	**0**
	GA	50		1	0	1			RH	17
	CT	78		1	0		1		DD	26
**5**	**0**		**0**	**0**	**0**	**0**	**0**	**0**	**0**	**0**
										0
**6**	**1**		**0**	**1**	**0**	**1**	**0**	**0**	**1**	**0**
	GA	406		1	0	1			AT	136,0
**7**	**5**		**0**	**1**	**4**	**3**	**2**	**0**	**5**	**0**
	AG	163		1	0	1			KE	55,0
	AT	249		0	1		1		AA	83
	TA	273		0	1		1		AA	91
	TA	375		0	1	1			HQ	125
	GC	406		0	1	1			AP	136,0
**8**	**2**		**1**	**1**	**1**	**0**	**1**	**0**	**2**	**0**
	AG	291		1	0		1		QQ	97
	INSERTION	462	1	0	1					154

**9**	**0**		**0**	**0**	**0**	**0**	**0**	**0**	**0**	**0**
										0
**10**	**0**		**0**	**0**	**0**	**0**	**0**	**0**	**0**	**0**
										0
**11**	**2**		**0**	**0**	**2**	**2**	**0**	**0**	**2**	**0**
	CA	223		0	1	1			QK	75,0
	CA	242		0	1	1			TN	81
**12**	**0**		**0**	**0**	**0**	**0**	**0**	**0**	**0**	**0**
										0
**13**	**1**		**0**	**1**	**0**	**1**	**0**	**0**	**1**	**0**
	GA	334		1	0	1			EK	112,0
**14**	**0**		**0**	**0**	**0**	**0**	**0**	**0**	**0**	**0**
										0
**15**	**0**		**0**	**0**	**0**	**0**	**0**	**0**	**0**	**0**
										0
**16**	**1**		**0**	**1**	**0**	**1**	**0**	**0**	**1**	**0**
	GA	445		1	0	1			VI	149,0
**17**	**1**		**0**	**1**	**0**	**1**	**0**	**0**	**1**	**0**
	CT	373		1	0	1			HY	125,0
**18**	**1**		**0**	**1**	**0**	**1**	**0**	**0**	**1**	**0**
	GA	50		1	0	1			RH	17
**19**	**0**		**0**	**0**	**0**	**0**	**0**	**0**	**0**	**0**
										0
**20**	**0**		**0**	**0**	**0**	**0**	**0**	**0**	**0**	**0**
										0
**21**	**1**		**0**	**1**	**0**	**0**	**1**	**0**	**0**	**0**
	GA	354		1	0		1		LL	118
**22**	**1**		**0**	**0**	**1**	**1**	**0**	**0**	**1**	**0**
	CA	515		0	1	1			AD	172
**23**	**1**		**0**	**1**	**0**	**1**	**0**	**0**	**1**	**0**

	CT	559		1	0	1			HY	187,0
**24**	**1**		**0**	**1**	**0**	**1**	**0**	**0**	**1**	**0**
	GA	232		1	0	1			DN	78,0
**25**	**0**		**0**	**0**	**0**	**0**	**0**	**0**	**0**	**0**
										0
**26**	**0**		**0**	**0**	**0**	**0**	**0**	**0**	**0**	**0**
										0
**27**	**0**		**0**	**0**	**0**	**0**	**0**	**0**	**0**	**0**
										0
**29**	**0**		**0**	**0**	**0**	**0**	**0**	**0**	**0**	**0**
										0
**30**	**3**		**0**	**1**	**2**	**1**	**2**	**0**	**3**	**0**
	TA	67		0	1	1			FI	23,0
	AT	456		0	1		1		KN	152
	AG	552		1	0		1		EE	184
**31**	**0**		**0**	**0**	**0**	**0**	**0**	**0**	**0**	**0**
										0
**32**	**1**		**0**	**1**	**0**	**1**	**0**	**0**	**1**	**0**
	CT	206		1	0	1			TI	69

TA	3
AT	2
CG	0
GC	1
TG	0
GT	0
GA	8
AG	3
CA	3
AC	0

TC	0
CT	4
INSERTION	1
DELETION	0

**Library sample**	**eIF4E med**									
**total mutations**	**78**		**0**	**60**	**18**	**62**	**14**	**2**		0
**Mutant ref**	**Mutations**	**nt Position**	**INDEL**	**Transition**	**Transversion**	**NS**	**S**	**STOP**	**aa substitution**	**aa Position**
**1**	**0**		**0**	**0**	**0**	**0**	**0**	**0**	**0**	**0**
										0
**2**	**1**		**0**	**0**	**1**	**1**	**0**	**0**	**1**	**0**
	GT	540		0	1	1			WC	180
**3**	**3**		**0**	**2**	**1**	**3**	**0**	**0**	**3**	**0**
	CT	138		1	0	1			SS	46
	CA	436		0	1	1			LI	146,0
	CT	551		1	0	1			AV	184
**4**	**5**		**0**	**4**	**1**	**4**	**1**	**0**	**5**	**0**
	GA	46		1	0	1			EK	16,0
	GA	123		1	0		1		TT	41
	AT	216		0	1	1			QH	72
	GA	373		1	0	1			VI	125,0
	AG	560		1	0	1			EG	187
**5**	**1**		**0**	**1**	**0**	**0**	**1**	**0**	**1**	**0**
	GA	168		1	0		1		EE	56
**6**	**3**		**0**	**3**	**0**	**2**	**1**	**0**	**3**	**0**
	CT	161		1	0	1			PL	54
	CT	301		1	0	1			PS	101,0
	CT	576		1	0		1		SS	192
**7**	**3**		**0**	**3**	**0**	**1**	**2**	**0**	**3**	**0**

	GA	431		1	0	1			SN	144
	TC	618		1	0		1		SS	206
	CT	654		1	0		1		LL	218
**8**	**3**		**0**	**2**	**1**	**2**	**0**	**1**	**3**	**0**
	GA	185		1	0			1	STOP	62
	GT	581		0	1	1			GV	194
	TC	625		1	0	1			FL	209,0
**9**	**1**		**0**	**0**	**1**	**1**	**0**	**0**	**1**	**0**
	AT	242		0	1	1			NI	81
**10**	**3**		**0**	**3**	**0**	**3**	**0**	**0**	**3**	**0**
	GA	190		1	0	1			DN	64,0
	GA	496		1	0	1			AT	166,0
	GA	508		1	0	1			VI	170,0
**11**	**0**		**0**	**0**	**0**	**0**	**0**	**0**	**0**	**0**
										0
**12**	**3**		**0**	**2**	**1**	**3**	**0**	**0**	**3**	**0**
	CT	260		1	0	1			TI	87
	CT	323		1	0	1			AV	108
	CA	422		0	1	1			SY	141
**13**	**1**		**0**	**1**	**0**	**0**	**1**	**0**	**1**	**0**
	GA	537		1	0		1		LL	179
**14**	**0**		**0**	**0**	**0**	**0**	**0**	**0**	**0**	**0**
										0
**15**	**8**		**0**	**6**	**2**	**6**	**2**	**0**	**8**	**0**
	CT	113		1	0	1			TI	38
	GA	217		1	0	1			AT	73,0
	GC	244		0	1	1			VL	82,0
	TC	293		1	0	1			IT	98
	TC	423		1	0		1		SS	141
	GA	585		1	0		1		KK	195

	GA	613		1	0	1			DN	205,0
	GC	643		0	1	1			AP	215,0
**16**	**3**		**0**	**3**	**0**	**1**	**2**	**0**	**3**	**0**
	CT	301		1	0	1			PS	101,0
	CT	432		1	0		1		SS	144
	TC	504		1	0		1		VV	168
**17**	**2**		**0**	**2**	**0**	**2**	**0**	**0**	**2**	**0**
	GA	70		1	0	1			EK	24,0
	CT	122		1	0	1			TM	41
**18**	**0**		**0**	**0**	**0**	**0**	**0**	**0**	**0**	**0**
										0,0
**19**	**2**		**0**	**2**	**0**	**2**	**0**	**0**	**2**	**0**
	CT	251		1	0	1			TI	84
	GA	316		1	0	1			VM	106,0
**20**	**1**		**0**	**1**	**0**	**1**	**0**	**0**	**1**	**0**
	GA	325		1	0	1			DN	109,0
**21**	**4**		**0**	**2**	**2**	**3**	**1**	**0**	**4**	**0**
	TA	120		0	1	1			DE	40
	AT	321		0	1		1		GG	107
	GA	431		1	0	1			SN	144
	GA	550		1	0	1			AT	184,0
**22**	**2**		**0**	**2**	**0**	**0**	**1**	**1**	**2**	**0**
	GA	185		1	0			1	STOP	62
	GA	309		1	0		1		KK	103
**23**	**2**		**0**	**1**	**1**	**2**	**0**	**0**	**2**	**0**
	AG	41		1	0	1			EG	14
	GC	230		0	1	1			ST	77
**24**	**0**		**0**	**0**	**0**	**0**	**0**	**0**	**0**	**0**
										0
**25**	**3**		**0**	**3**	**0**	**2**	**1**	**0**	**3**	**0**

	GA	85		1	0	1			VI	29,0
	GA	379		1	0	1			AT	127,0
	TC	423		1	0		1		SS	141
**26**	**5**		**0**	**2**	**3**	**5**	**0**	**0**	**5**	**0**
	CT	125		1	0	1			TM	42
	CT	251		1	0	1			TI	84
	CA	281		0	1	1			AD	94
	TA	572		0	1	1			VD	191
	AT	647		0	1	1			KM	216
**27**	**1**		**0**	**0**	**1**	**1**	**0**	**0**	**1**	**0**
	CG	343		0	1	1			HD	115,0
**28**	**3**		**0**	**2**	**1**	**3**	**0**	**0**	**3**	**0**
	AC	108		0	1	1			ED	36
	CT	169		1	0	1			HY	57,0
	GA	227		1	0	1			GD	76
**29**	**2**		**0**	**0**	**2**	**1**	**1**	**0**	**2**	**0**
	AC	50		0	1	1			KI	17
	TA	102		0	1		1		II	34
**30**	**5**		**0**	**5**	**0**	**5**	**0**	**0**	**5**	**0**
	CT	65		1	0	1			AV	22
	TC	253		1	0	1			FL	85,0
	GA	305		1	0	1			SN	102
	CT	497		1	0	1			AV	166
	GA	520		1	0	1			GR	174,0
**31**	**5**		**0**	**5**	**0**	**5**	**0**	**0**	**5**	**0**
	GA	79		1	0	1			DN	27,0
	CT	161		1	0	1			PL	54
	GA	313		1	0	1			VI	105,0
	CT	428		1	0	1			TI	143
	CT	443		1	0	1			TM	148

**32**	**3**		**0**	**3**	**0**	**3**	**0**	**0**	**3**	**0**
	CT	169		1	0	1			HY	57,0
	GA	502		1	0	1			VI	168,0
	AG	646		1	0	1			KE	216,0

TA	3
AT	4
CG	1
GC	3
TG	0
GT	2
GA	28
AG	3
CA	3
AC	2
TC	7
CT	22

INSERTION	0
DELETION	0

Library sample **VPg MED**

**total mutations**	**66**		**4**	**31**	**36**	**41**	**21**	**0**		0
**Mutant ref**	**Mutations**	**nt Position**	**INDEL**	**Transition**	**Transversion**	**NS**	**S**	**STOP**	**aa substitution**	**aa Position**
**312**	**2**		**0**	**2**	**0**	**1**	**1**	**0**	**2**	**0**
	GA	159		1	0	1			MI	53,0
	TC	198		1	0		1		FF	66,0

**311**	**2**		**0**	**1**	**1**	**1**	**1**	**0**	**2**	**0**
	TA	278		0	1	1			IN	93,0
	TC	421		1	0		1		LL	141,0
**310**	**2**		**1**	**0**	**2**	**1**	**0**	**0**	**2**	**0**
	INSERTION	223	1	0	1					74,3
	TA	300		0	1	1			FL	100,0
**308**	**2**		**0**	**1**	**1**	**2**	**0**	**0**	**2**	**0**
	GA	88		1	0	1			DN	30,0
	AT	431		0	1	1			HL	144,0
**307**	**4**		**0**	**2**	**2**	**1**	**3**	**0**	**4**	**0**
	AG	258		1	0		1		EE	86,0
	GT	366		0	1		1		TT	122,0
	GA	414		1	0		1		KK	138,0
	GT	509		0	1	1			GV	170,0
**305**	**3**		**0**	**2**	**1**	**1**	**2**	**0**	**3**	**0**
	TC	39		1	0		1		FF	13,0
	CA	186		0	1	1			NK	62,0
	GA	309		1	0		1		VV	103,0
**304**	**5**		**0**	**1**	**4**	**3**	**2**	**0**	**5**	**0**
	GT	154		0	1	1			GC	52,0
	TA	243		0	1		1		TT	81,0
	GT	318		0	1	1			KN	106,0
	TA	425		0	1	1			MK	142,0
	GA	498		1	0		1		LL	166,0
**303**	**4**		**0**	**1**	**3**	**4**	**0**	**0**	**4**	**0**
	CA	49		0	1	1			RS	17,0
	AG	145		1	0	1			TA	49,0
	GT	170		0	1	1			SI	57,0
	TA	467		0	1	1			IN	156,0
**302**	**3**		**1**	**2**	**1**	**2**	**0**	**0**	**3**	**0**

	TC	95		1	0	1			IT	32,0
	AG	290		1	0	1			QR	97,0
	INSERTION	546	1	0	1					182,0
**301**	**3**		**0**	**0**	**3**	**3**	**0**	**0**	**3**	**0**
	TA	254		0	1	1			IK	85,0
	AT	473		0	1	1			KI	158,0
	AT	533		0	1	1			KM	178,0
**330**	**6**		**0**	**2**	**4**	**5**	**1**	**0**	**6**	**0**
	TA	234		0	1	1			DE	78,0
	AT	367		0	1	1			SC	123,0
	TA	378		0	1		1		AA	126,0
	AG	380		1	0	1			YC	127,0
	AT	431		0	1	1			HL	144,0
	GA	535		1	0	1			DN	179,0
**329**	**5**		**0**	**4**	**1**	**3**	**2**	**0**	**5**	**0**
	CT	186		1	0		1		NN	62,0
	AG	313		1	0	1			RG	105,0
	AT	326		0	1	1			EV	109,0
	GA	366		1	0		1		TT	122,0
	AG	413		1	0	1			KR	138,0
**328**	**3**		**0**	**2**	**1**	**2**	**1**	**0**	**3**	**0**
	TA	108		0	1	1			FL	36,0
	GA	354		1	0		1		LL	118,0
	CT	365		1	0	1			TM	122,0
**326**	**4**		**0**	**1**	**3**	**3**	**1**	**0**	**4**	**0**
	CA	147		0	1		1		TT	49,0
	CG	149		0	1	1			TR	50,0
	TA	268		0	1	1			YN	90,0
	CT	343		1	0	1			PS	115,0
**325**	**0**		**0**	**0**	**1**	**0**	**0**	**0**	**0**	**0**

				0	1					
**324**	**5**		**1**	**3**	**2**	**2**	**2**	**0**	**5**	**0**
	INSERTION	199	1	0	1					66,3
	GC	247		0	1	1			AP	83,0
	AG	438		1	0		1		PP	146,0
	CT	496		1	0		1		LL	166,0
	AG	544		1	0	1			KE	182,0
**323**	**3**		**0**	**1**	**2**	**3**	**0**	**0**	**3**	**0**
	GT	122		0	1	1			RM	41,0
	AT	362		0	1	1			NI	121,0
	CT	511		1	0	1			PS	171,0
**322**	**2**		**1**	**0**	**2**	**1**	**0**	**0**	**2**	**0**
	TA	211		0	1	1			YN	71,0
	DELETION	411	1	0	1					137,0
**321**	**6**		**0**	**5**	**1**	**2**	**4**	**0**	**6**	**0**
	AG	93		1	0		1		TT	31,0
	TC	218		1	0	1			FS	73,0
	CT	402		1	0		1		DD	134,0
	AG	405		1	0		1		KK	135,0
	CG	428		0	1	1			PR	143,0
	GA	489		1	0		1		EE	163,0
**320**	**2**		**0**	**1**	**1**	**1**	**1**	**0**	**2**	**0**
	TA	360		0	1	1			SR	120,0
	AG	405		1	0		1		KK	135,0

TA	12
AT	7
CG	2
GC	1
TG	0

GT	6
GA	9
AG	11
CA	3
AC	0
TC	5
CT	6
INSERTION	3
DELETION	1

**Library sample**	**eIF4E HIGH**									
**total mutations**	**223**	**2**	**11**	**93**	**77**	**155**	**44**	**13**		0
**Mutant ref**	**Mutations**	**nt Position**	**INDEL**	**Transition**	**Transversion**	**NS**	**S**	**STOP**	**aa substitution**	**aa Position**
**1**	**5**		**0**	**3**	**2**	**4**	**1**	**0**	**5**	**0**
	AT	67		0	1	1			NY	23,0
	GA	126		1	0		1		TT	42
	TA	490		0	1	1			CS	164,0
	CT	497		1	0	1			AV	166
	GA	617		1	0	1			SN	206
**1'**	**13**		**1**	**3**	**3**	**8**	**4**	**0**	**13**	**0**
	AT	107		0	1	1			EV	36
	AT	112		0	1	1			TS	28,0
	AG	139		1	0	1			KE	47,0
	GA	148		1	0	1			AT	50,0
	CT	157		1	0	1			HY	53,0

	CG	221		0	1	1			AG	74
	DELETION	238	1							80,0
	TA	279		0	1		1		GG	93
	GT	379		0	1	1			AS	127,0
	GA	444		1	0		1		TT	148
	TC	450		1	0		1		LL	150
	GA	502		1	0	1			VI	168,0
	GT	603		0	1		1		LL	201
**2'**	**9**		**0**	**3**	**2**	**8**	**1**	**0**	**9**	**0**
	CA	44		0	1	1			AD	15
	AG	124		1	0	1			TA	42,0
	AT	286		0	1	1			NY	96,0
	GA	404		1	0	1			SN	135
	CT	410		1	0	1			SL	137
	AT	480		0	1	1			ED	160
	GT	499		0	1	1			VL	167,0
	TC	633		1	0		1		FF	211
	AT	677		0	1	1			YF	226
**3**	**4**		**0**	**5**	**4**	**4**	**0**	**0**	**4**	**0**
	AT	99		0	1	1			ED	33
	AG	154		1	0	1			KE	52,0
	TA	437		0	1	1			LQ	146
	AT	486		0	1	1			ED	162
**3'**	**9**		**1**	**4**	**1**	**3**	**4**	**1**	**9**	**0**
	CT	231		1	0		1		SS	77
	AG	241		1	0	1			ND	81,0
	AT	267		0	1	1			ED	89
	GA	309		1	0		1		KK	103
	GA	342		1	0		1		KK	114
	AT	358		0	1			1	STOP	120,0

	INSERTION	400	1							134,0
	CT	612		1	0		1		SS	204
	AG	628		1	0	1			IV	210,0
**4**	**6**		**0**	**2**	**3**	**4**	**1**	**1**	**6**	**0**
	AG	151		1	0	1			TA	51,0
	TG	310		0	1	1			LV	104,0
	TA	509		0	1	1			VD	170
	AG	660		1	0		1		RR	220
	TA	663		0	1	1			NK	221
	CG	678		0	1			1	STOP	226
**4'**	**0**									
										0
**5**	**6**		**0**	**4**	**1**	**5**	**1**	**0**	**6**	**0**
	CT	125		1	0	1			TM	42
	CG	258		0	1		1		SS	86
	AG	382		1	0	1			ND	128,0
	GA	386		1	0	1			GE	129
	GA	402		1	0	1			MI	134
	TA	426		0	1	1			DE	142
**5'**	**6**		**0**	**3**	**2**	**5**	**1**	**0**	**6**	**0**
	GA	106		1	0	1			EK	36,0
	CA	113		0	1	1			TN	38
	GC	190		0	1	1			DH	64,0
	GA	404		1	0	1			SN	135
	GA	444		1	0		1		TT	148
	AT	480		0	1	1			ED	160
**6**	**5**		**0**	**1**	**4**	**4**	**1**	**0**	**5**	**0**
	GT	48		0	1	1			ED	16
	AT	61		0	1	1			NY	21,0
	GT	402		0	1	1			MI	134

	AT	485		0	1	1			EV	162
	TC	672		1	0		1		NN	224
**6'**	**5**		**0**	**0**	**5**	**4**	**1**	**0**	**5**	**0**
	AT	178		0	1	1			TS	60,0
	TA	184		0	1	1			WR	62,0
	GC	316		0	1	1			VL	106,0
	AT	348		0	1	1			KN	116
	AT	357		0	1		1		PP	119
**7**	**0**									
										0
**7'**	**8**		**1**	**3**	**1**	**6**	**0**	**1**	**8**	**0**
	GT	106		0	1			1	STOP	36,0
	CT	128		1	0	1			SL	43
	DELETION	155	1							52
	GA	220		1	0	1			AT	74,0
	GA	278		1	0	1			GD	93
	AG	365		1	0	1			EG	122
	AT	560		0	1	1			EV	187
	CT	566		1	0	1			AV	189
**8**	**5**		**0**	**3**	**2**	**4**	**1**	**0**	**5**	**0**
	TA	81		0	1	1			DE	27
	CA	214		0	1	1			QK	72,0
	CT	240		1	0		1		RR	80
	GA	424		1	0	1			DN	142,0
	TC	572		1	0	1			VA	191
**8'**	**6**		**0**	**2**	**3**	**3**	**3**	**0**	**6**	**0**
	TA	189		0	1	1			FL	63
	CT	300		1	0		1		HH	100
	GC	316		0	1	1			VL	106,0
	TC	492		1	0		1		CC	164

	AT	501		0	1		1		VV	167
	TG	535		0	1	1			LV	179,0
**10**	**5**		**0**	**1**	**4**	**3**	**1**	**1**	**5**	**0**
	GA	224		1	0			1	STOP	75
	AT	382		0	1	1			NY	128,0
	TA	465		0	1	1			HQ	155
	AT	505		0	1	1			SC	169,0
	AT	645		0	1		1		AA	215
**11**	**6**		**0**	**0**	**5**	**6**	**0**	**0**	**6**	**0**
	AT	143		0	1	1			EV	48
	GT	424		0	1	1			DY	142,0
	CG	445		0	1	1			LV	149,0
	GC	508		0	1	1			VL	170,0
	GC	637		0	1	1			DH	213,0
	GA	650		1	0	1			RK	217
**12**	**6**		**0**	**3**	**2**	**3**	**2**	**1**	**6**	**0**
	GT	37		0	1	1			DY	13,0
	TC	270		1	0		1		DD	90
	CA	410		0	1			1	STOP	137
	AG	522		1	0		1		GG	174
	TC	599		1	0	1			FS	200
	AG	671		1	0	1			NS	224
**13**	**5**		**0**	**3**	**2**	**3**	**1**	**1**	**5**	**0**
	GA	129		1	0		1		SS	43
	GA	224		1	0			1	STOP	75
	GA	506		1	0	1			SN	169
	CG	563		0	1	1			TR	188
	TA	599		0	1	1			FY	200
**14**	**6**		**0**	**2**	**3**	**5**	**1**	**0**	**6**	**0**
	AC	61		0	1	1			NH	21,0

	CT	152		1	0	1			TI	51
	TC	172		1	0	1			SP	58,0
	AT	267		0	1	1			ED	89
	GT	590		0	1	1			WL	197
	CT	636		1	0		1		HH	212
**15**	**13**		**1**	**1**	**4**	**7**	**3**	**2**	**13**	**0**
	AT	150		0	1		1		AA	50
	TC	237		1	0		1		LL	79
	AT	332		0	1	1			HL	111
	TA	441		0	1			1	STOP	147
	CG	497		0	1	1			AG	166
	GT	514		0	1	1			GC	172,0
	DELETION	524	1							175
	GT	564		0	1		1		TT	188
	AT	577		0	1	1			IF	193,0
	AG	593		1	0	1			KR	198
	AC	641		0	1	1			DA	214
	AT	646		0	1			1	STOP	216,0
	GC	682		0	1	1			VL	228,0
**16**	**11**		**0**	**3**	**2**	**6**	**3**	**2**	**11**	**0**
	AG	112		1	0	1			TA	37
	GA	156		1	0		1		KK	52
	CA	233		0	1			1	STOP	78
	TC	252		1	0		1		TT	84
	CA	356		0	1	1			PQ	119
	GA	389		1	0	1			GE	130
	AG	430		1	0	1			SG	144,0
	CA	463		0	1	1			HN	155,0
	AT	592		0	1			1	STOP	198,0
	CT	615		1	0		1		DD	205

	AT	656		0	1	1			DV	219
**19**	**3**		**0**	**6**	**3**	**3**	**0**	**0**	**3**	**0**
	GT	313		0	1	1			VF	105,0
	GT	591		0	1	1			WC	197
	AG	635		1	0	1			HR	212
**20**	**8**		**1**	**4**	**1**	**5**	**1**	**1**	**8**	**0**
	AG	119		1	0	1			DG	40
	AG	139		1	0	1			KE	47,0
	TC	329		1	0	1			LS	110
	TA	350		0	1	1			IN	117
	GA	354		1	0		1		EE	118
	GA	395		1	0			1	STOP	132
	CA	554		0	1	1			AE	185
	DELETION	647	1							216
**21**	**5**		**0**	**3**	**2**	**4**	**1**	**0**	**5**	**0**
	TG	120		0	1	1			DE	40
	TA	270		0	1	1			DE	90
	GA	386		1	0	1			GE	129
	TC	421		1	0	1			SP	141,0
	CT	657		1	0		1		DD	219
**22**	**6**		**0**	**4**	**1**	**6**	**0**	**0**	**6**	**0**
	AT	41		0	1	1			EV	14
	GA	64		1	0	1			AT	22,0
	CT	152		1	0	1			TI	51
	TC	253		1	0	1			FL	85,0
	TC	274		1	0	1			WR	92,0
	GT	648		0	1	1			KN	216
**23**	**5**		**1**	**1**	**3**	**3**	**0**	**1**	**5**	**0**
	CT	74		1	0	1			AV	25
	TA	333		0	1	1			HQ	111

	CA	410		0	1			1	STOP	137
	CA	576		0	1	1			SR	192
	DELETION	633	1							211
**24**	**11**		**1**	**5**	**0**	**8**	**1**	**1**	**11**	**0**
	AG	154		1	0	1			KE	52,0
	CT	196		1	0	1			PS	66,0
	TC	219		1	0		1		AA	73
	GA	220		1	0	1			AT	74,0
	GA	363		1	0			1	STOP	121
	AT	365		0	1	1			EV	122
	DELETION	413	1							138
	AT	529		0	1	1			IL	177,0
	AT	548		0	1	1			NI	183
	AT	577		0	1	1			IF	193,0
	CT	673		1	0	1			RC	225,0
**27**	**11**		**1**	**4**	**1**	**8**	**2**	**0**	**11**	**0**
	CA	160		0	1	1			PT	54,0
	TC	163		1	0		1		LL	55,0
	CT	281		1	0	1			AV	94
	CT	463		1	0	1			HY	155,0
	GA	514		1	0	1			GS	172,0
	CT	551		1	0	1			AV	184
	GC	565		0	1	1			AP	189,0
	DELETION	647	1							216
	TA	653		0	1	1			LH	218
	CT	660		1	0		1		DD	220
	AT	657		0	1	1			RS	219
**28**	**6**		**0**	**2**	**3**	**6**	**0**	**0**	**6**	**0**
	AG	131		1	0	1			YC	44
	TA	270		0	1	1			DE	90

	AT	383		0	1	1			NI	128
	GC	416		0	1	1			GA	139
	TC	470		1	0	1			FS	157
	CT	497		1	0	1			AV	166
**29**	**3**		**1**	**5**	**3**	**0**	**2**	**0**	**3**	**0**
	GA	234		1	0		1		SS	78
	GA	585		1	0		1		KK	195
	DELETION	647	1							216
**30**	**10**	**2**	**2**	**3**	**2**	**4**	**4**	**0**	**10**	**-2**
	GT	51		0	1	1			KN	17
	AG	55		1	0	1			KE	19,0
	GA	72		1	0		1		EE	24
	TA	86		0	1	1			VD	29
	GA	278		1	0	1			GD	93
	AG	324		1	0		1		AA	108
	TA	489		0	1		1		II	163
	DELETION	529	1							177,0
	TC	549		1	0		1		NN	183
	DELETION	647	1							216
**31**	**8**		**0**	**3**	**2**	**6**	**2**	**0**	**8**	**0**
	AG	119		1	0	1			DG	40
	CT	157		1	0	1			HY	53,0
	CA	295		0	1	1			HN	99,0
	AT	398		0	1	1			KI	133
	TC	489		1	0		1		II	163
	CT	627		1	0		1		FF	209
	AT	647		0	1	1			KM	216
	CA	652		0	1	1			LI	218,0
**32**	**8**		**0**	**4**	**1**	**7**	**1**	**0**	**8**	**0**
	GA	88		1	0	1			EK	30,0

GA	230	1	0	1		SN	77
GA	244	1	0	1		VI	82,0
CG	443	0	1	1		TR	148
TC	534	1	0		1	SS	178
CG	566	0	1	1		AG	189
GA	622	1	0	1		GS	208,0
TA	642	0	1	1		DE	214

TA	20
AT	38
CG	8
GC	8
TG	3
GT	15
GA	38
AG	22
CA	13
AC	2
TC	20
CT	25
INSERTION	1
DELETION	10

**Library sample**	**VPg HIGH**								
**total mutations**	**229**	**0**	**12**	**120**	**107**	**156**	**52**	**9**	0

Mutant ref	Mutations	nt Position	INDEL	Transition	Transversion	NS	S	STOP	aa substitution	aa Position
**1**	**8**		**0**	**6**	**2**	**5**	**3**	**0**	**8**	**0**
	GA	65		1	0	1			GD	22
	AG	140		1	0	1			KR	47
	TA	152		0	1	1			VD	51
	TC	156		1	0		1		GG	52
	CT	162		1	0		1		GG	54
	GC	414		0	1	1			KN	138
	TC	475		1	0	1			FL	159,0
	GA	489		1	0		1		EE	163
**2**	**7**		**2**	**1**	**6**	**3**	**1**	**1**	**7**	**0**
	DELETION	155	1	0	1					52
	AT	195		0	1		1		GG	65
	AG	313		1	0	1			RG	105,0
	CA	369		0	1	1			SR	123
	AT	370		0	1	1			IL	124,0
	DELETION	434	1	0	1					145
	TA	450		0	1			1	STOP	150
**3**	**5**		**2**	**1**	**4**	**3**	**0**	**0**	**5**	**0**
	DELETION	65	1	0	1					22
	AT	258		0	1	1			ED	86
	DELETION	279	1	0	1					93
	AG	372		1	0	1			IM	124
	GT	386		0	1	1			RM	129
**4**	**5**		**0**	**3**	**2**	**5**	**0**	**0**	**5**	**0**
	GC	265		0	1	1			VL	89,0
	TC	383		1	0	1			FS	128
	AG	473		1	0	1			KR	158
	CA	479		0	1	1			PH	160
	AG	527		1	0	1			NS	176

**5**	**5**		**0**	**3**	**2**	**4**	**1**	**0**	**5**	**0**
	GA	376		1	0	1			AT	126,0
	TA	378		0	1	1			AT	126
	GC	504		0	1	1			QH	168
	GA	517		1	0	1			VM	173,0
	TC	561		1	0		1		HH	187
**6**	**6**		**0**	**4**	**2**	**5**	**1**	**0**	**6**	**0**
	GA	65		1	0	1			GD	22
	AC	83		0	1	1			NT	28
	CT	149		1	0	1			TI	50
	TC	234		1	0		1		DD	78
	CT	242		1	0	1			TI	81
	GC	256		0	1	1			EQ	86,0
**7**	**11**		**0**	**5**	**6**	**6**	**3**	**2**	**11**	**0**
	AT	124		0	1			1	STOP	42,0
	AT	139		0	1			1	STOP	47,0
	CT	180		1	0		1		FF	60
	AT	204		0	1		1		PP	68
	CT	267		1	0		1		VV	89
	GC	315		0	1	1			RS	105
	AT	326		0	1	1			EV	109
	GC	340		0	1	1			ER	114,0
	AG	341		1	0	1			ER	114
	GA	469		1	0	1			AT	157,0
	AG	548		1	0	1			QR	183
**8**	**8**		**2**	**5**	**3**	**3**	**3**	**0**	**8**	**0**
	GA	57		1	0		1		KK	19
	DELETION	110	1	0	1					37
	TA	118		0	1	1			YN	40,0
	AG	133		1	0	1			KE	45,0

	CT	180		1	0		1		FF	60
	TC	196		1	0	1			FL	66,0
	DELETION	223	1	0	1					75,0
	CT	240		1	0		1		FF	80
**9**	**6**		**0**	**4**	**2**	**5**	**1**	**0**	**6**	**0**
	CT	42		1	0		1		RR	14
	CT	47		1	0	1			AV	16
	GA	52		1	0	1			DN	18,0
	GT	281		0	1	1			RM	94
	GA	400		1	0	1			DN	134,0
	AC	405		0	1	1			KN	135
**10**	**4**		**0**	**3**	**1**	**3**	**1**	**0**	**4**	**0**
	GA	154		1	0	1			GS	52,0
	CT	377		1	0	1			AV	126
	AT	494		0	1	1			EV	165
	AG	552		1	0		1		EE	184
**14**	**8**		**0**	**2**	**6**	**7**	**1**	**0**	**8**	**0**
	GA	99		1	0		1		EE	33
	GC	155		0	1	1			GA	52
	GT	176		0	1	1			RI	59
	GT	368		0	1	1			SI	123
	AT	405		0	1	1			KN	135
	AC	466		0	1	1			IL	156,0
	TC	491		1	0	1			FS	164
	CG	512		0	1	1			PR	171
**15**	**7**		**0**	**1**	**6**	**6**	**1**	**0**	**7**	**0**
	AT	80		0	1	1			NI	27
	AT	225		0	1	1			QH	75
	CT	248		1	0	1			AV	83
	CA	351		0	1		1		AA	117

	GC	386		0	1	1			RT	129
	GC	485		0	1	1			RT	162
	AT	499		0	1	1			RW	167,0
**16**	**14**		**0**	**8**	**6**	**8**	**5**	**1**	**14**	**0**
	AT	44		0	1	1			HL	15
	AT	121		0	1	1			RW	41,0
	CA	147		0	1		1		TT	49
	CT	242		1	0	1			TI	81
	AT	316		0	1			1	STOP	106,0
	GA	376		1	0	1			AI	126,0
	CT	377		1	0	1			AI	126
	TC	399		1	0		1		SS	133
	TC	425		1	0	1			MT	142
	AT	431		0	1	1			HL	144
	CT	453		1	0		1		DD	151
	AG	486		1	0		1		RR	162
	TA	491		0	1	1			FY	164
	AG	552		1	0		1		EE	184
**17**	**9**		**1**	**5**	**3**	**7**	**1**	**0**	**9**	**0**
	AT	72		0	1	1			ED	24
	AG	96		1	0	1			IM	32
	AG	262		1	0	1			ND	88,0
	TG	393		0	1	1			DE	131
	TC	409		1	0		1		LL	137,0
	AG	461		1	0	1			NS	154
	GA	469		1	0	1			AT	157,0
	CG	511		0	1	1			PA	171,0
	**DELETION**	535	1	0						179,0
**18**	**12**		**1**	**9**	**2**	**9**	**1**	**1**	**12**	**0**
	GA	41		1	0	1			RH	14

	CT	42		1	0	1			RH	14
	AT	86		0	1	1			DV	29
	AG	157		1	0	1			MV	53,0
	GC	170		0	1	1			ST	57
	TC	211		1	0	1			YH	71,0
	DELETION	222	1	0						74
	TC	360		1	0		1		SS	120
	GA	400		1	0	1			DN	134,0
	CT	402		1	0	1			DN	134
	AG	434		1	0	1			NS	145
	CT	502		1	0			1	STOP	168,0
**19**	**11**		**0**	**8**	**3**	**8**	**3**	**0**	**11**	**0**
	TC	107		1	0	1			FS	36
	GA	154		1	0	1			GS	52,0
	GA	259		1	0	1			EK	87,0
	GA	309		1	0		1		VV	103
	TA	320		0	1	1			MK	107
	AG	342		1	0		1		EE	114
	AT	361		0	1	1			NY	121,0
	CT	365		1	0	1			TM	122
	CT	402		1	0		1		DD	134
	AT	455		0	1	1			KI	152
	GA	508		1	0	1			GR	170,0
**20**	**7**		**0**	**4**	**3**	**7**	**0**	**0**	**7**	**0**
	TA	103		0	1	1			FI	35,0
	GC	155		0	1	1			GA	52
	GA	245		1	0	1			GE	82
	GA	331		1	0	1			DN	111,0
	TC	410		1	0	1			LS	137
	CA	428		0	1	1			PQ	143

	AG	473		1	0	1			KR	158
**21**	**5**		**0**	**5**	**0**	**2**	**2**	**1**	**5**	**0**
	GA	265		1	0	1			VI	89,0
	CT	344		1	0	1			PL	115
	GA	396		1	0			1	STOP	132
	GA	498		1	0		1		LL	166
	TC	516		1	0		1		AA	172
**22**	**11**		**0**	**5**	**6**	**10**	**1**	**0**	**11**	**0**
	GT	61		0	1	1			AS	21,0
	CT	120		1	0		1		YY	40
	GC	155		0	1	1			GA	52
	AG	233		1	0	1			DG	78
	AG	284		1	0	1			DG	95
	TA	300		0	1	1			FL	100
	AG	305		0	1	1			EG	102
	CT	377		1	0	1			AV	126
	CG	430		1	0	1			HD	144,0
	AT	466		0	1	1			IF	156,0
	TA	497		0	1	1			LQ	166
**23**	**8**		**0**	**6**	**2**	**6**	**2**	**0**	**8**	**0**
	TC	84		1	0		1		NN	28
	AG	133		1	0	1			KE	45,0
	CT	146		1	0	1			TI	49
	AG	204		1	0		1		PP	68
	AT	205		0	1	1			TS	69,0
	GT	349		0	1	1			AS	117,0
	AG	460		1	0	1			ND	154,0
	GA	464		1	0	1			GD	155
**24**	**13**		**0**	**7**	**6**	**8**	**4**	**1**	**13**	**0**
	GC	123		0	1	1			RS	41

	AT	191		0	1	1			YF	64
	CA	228		0	1	1			FL	76
	TC	270		1	0		1		YY	90
	TC	300		1	0		1		FF	100
	CT	344		1	0	1			PL	115
	GA	349		1	0	1			AT	117,0
	GA	366		1	0		1		TT	122
	CG	377		0	1	1			AG	126
	GA	418		1	0	1			DN	140,0
	AT	532		0	1			1	STOP	178,0
	AG	536		1	0	1			DG	179
	AT	540		0	1		1		II	180
**25**	**8**		**0**	**5**	**3**	**4**	**4**	**0**	**8**	**0**
	AT	96		0	1		1		II	32
	AG	216		1	0		1		SS	72
	CT	219		1	0		1		FF	73
	AG	269		1	0	1			YC	90
	CT	377		1	0	1			AV	126
	GC	444		0	1	1			KN	148
	AG	459		1	0		1		TT	153
	AT	538		0	1	1			IL	180,0
**26**	**11**		**0**	**7**	**4**	**5**	**5**	**1**	**11**	**0**
	AT	34		0	1			1	STOP	12,0
	CT	78		1	0		1		DD	26
	TC	114		1	0		1		SS	38
	TA	198		0	1	1			FL	66
	GA	247		1	0	1			AT	83,0
	AG	312		1	0		1		RR	104
	TC	323		1	0	1			VA	108
	GT	331		0	1	1			DY	111,0

	GA	411		1	0		1		LL	137
	GA	517		1	0	1			VM	173,0
	AT	540		0	1		1		II	180
**27**	**5**		**2**	**2**	**3**	**3**	**0**	**0**	**5**	**0**
	INSERTION	103	1	0	1					35,0
	DELETION	225	1	0	1					75
	GT	349		0	1	1			AS	117,0
	CT	365		1	0	1			TM	122
	GA	529		1	0	1			VM	177,0
**28**	**7**		**0**	**2**	**5**	**5**	**2**	**0**	**7**	**0**
	GA	60		1	0		1		RR	20
	GA	65		1	0	1			GD	22
	GT	307		0	1	1			VL	103,0
	TG	323		0	1	1			VG	108
	CG	369		0	1	1			SR	123
	AC	429		0	1		1		PP	143
	GT	500		0	1	1			RM	167
**29**	**11**		**1**	**2**	**9**	**8**	**2**	**0**	**11**	**0**
	TA	48		0	1		1		AA	16
	GT	57		0	1	1			KN	19
	GT	76		0	1	1			DY	26,0
	GC	97		0	1	1			EQ	33,0
	GC	155		0	1	1			GA	52
	TC	179		1	0	1			FS	60
	TC	264		1	0		1		NN	88
	CA	272		0	1	1			AD	91
	AT	317		0	1	1			KM	106
	DELETION	351	1	0	1					117
	TA	539		0	1	1			IK	180
**30**	**7**		**0**	**4**	**3**	**3**	**3**	**1**	**7**	**0**

	CG	62		0	1	1			AG	21
	TC	84		1	0		1		NN	28
	CT	240		1	0		1		LL	80
	TA	266		0	1	1			VD	89
	AT	341		0	1	1			EV	114
	CT	369		1	0		1		SS	123
	GA	396		1	0			1	STOP	132
**31**	**4**		**1**	**1**	**3**	**3**	**0**	**0**	**4**	**0**
	DELETION	141	1	0	1					47
	GA	161		1	0	1			GD	54
	CG	272		0	1	1			AG	91
	GC	354		0	1	1			LF	118
**33**	**6**		**0**	**2**	**4**	**5**	**1**	**0**	**6**	**0**
	AT	125		0	1	1			KM	42
	CG	149		0	1	1			TR	50
	TG	190		0	1	1			YD	64,0
	GA	322		1	0	1			VI	108,0
	AT	404		0	1	1			KI	135
	TC	408		1	0		1		AA	136

TA	13
AT	33
CG	8
GC	17
TG	3
GT	12
GA	37
AG	29
CA	7
