# Supplementary material for: Comparative analysis of mutational robustness of the intrinsically disordered viral protein VPg and of its interactor eIF4E
Source: PLoS One. 2019 Feb 14;14(2):e0211725. doi: 10.1371/journal.pone.0211725 (PMC6375565; doi:10.1371/journal.pone.0211725)
Supplement: S1 Code — (PDF) [file pone.0211725.s007.pdf]

S1 Code: Rcode for codon volatility calculation

Codon volatility calculation

The likelihood that a random point mutation within each codon gives either a synonymous codon (file CdnSyn.csv) or a stop codon (file Cdnstp.csv) was calculated. Protein codon files: VPg.csv; eIF4E: 4E.csv. Likelihoods obtained for each codon were averaged over the whole protein sequence (file eIF4E and VPg volatility.xlsx). A t test was used to assess if the two values (VPg and eIF4E volatilities) were significantly different.

R script for codon volatility calculation

```
codonsyn<-read.table("CdnSyn.csv",sep= ';' ,header=TRUE)        # File containing for each codon its volatility level under the simpliest model of mutation (syn).
codonstp<-read.table(" Cdnstp.csv",sep= ';' ,header=TRUE)        # File containing for each codon its probability under simpliest model of mutation (stop).
seq = read.table("4E.csv",sep= ' ' ,header=FALSE)                      # File containing each codon of the tested protein (sequence file of the protein; for this work : eIF4E, file: 4E.csv; VPg, file: VPg.csv). )

# Allocation of synonymous substitution for each codon of the tested protein
l = dim(seq)[1] All1=NULL
for (a in 1:l) {  q =
as.character(seq[a,1])
score = codonsyn[codonsyn[,1] == q,2]
Chge = matrix(c(q,as.character(score)), nrow=1, ncol=2, byrow=FALSE)
All1 <-rbind(All1,Chge)
                    }

# Allocation of stop substitution for each codon of the tested protein
l = dim(seq)[1] All2 =NULL
for (a in 1:l) {
  q = as.character(seq[a,1])
  score = codonstp[codonstp[,1] == q,2]
  Chge = matrix(c(q,as.character(score)), nrow=1, ncol=2, byrow=FALSE)
  All2 <-rbind(All2,Chge)
                    }
```
